# Music and health: what interventions for what results?

**DOI:** 10.3389/fpsyg.2015.00230

**Published:** 2015-03-02

**Authors:** Alfredo Raglio, Osmano Oasi

**Affiliations:** ^1^Department of Public Health, Experimental and Forensic Medicine, University of PaviaPavia, Italy; ^2^Department of Psychology, Catholic University of MilanMilan, Italy

**Keywords:** music, music therapy, evidence-based approach, health

For several decades, music has been used more and more frequently and consciously as a mean of care to reduce or stabilize symptoms and/or complications arising therefrom. This has been the case with several diseases, including chronic and degenerative ones (in psychiatry, child neuropsychiatry, neurology, oncology, palliative care, etc.) (Gold et al., 2004; Särkämö et al., 2008; Bradt et al., 2011; Erkkilä et al., 2011; Mössler et al., 2011; Raglio et al., 2012; Bradt and Dileo, 2014) and in contexts in which the symptoms are just momentary (e.g., in hospitals before surgical interventions in order to reduce anxiety and stress, or to reduce the perception of pain during invasive medical procedures) (Bradt et al., 2013; Cepeda et al., 2013). Indeed, music also gives pleasure, promotes well-being, facilitates the expression and regulation of emotions and improves communication and relationships between individuals (Hillecke et al., 2005). Numerous systematic reviews of the literature (including many Cochrane Reviews), randomized controlled or controlled clinical trials and qualitative studies, all show the significant results that come with the therapeutic use of music.

The basis underlying the therapeutic potential of music are to be considered in relation to the extensive action which music itself exerts on the brain at the cortical level but also at the limbic and paralimbic ones (Koelsch, 2009; Levitin and Tirovolas, 2009). Music and sound also affect vital signs and neurochemical systems (such as those of dopamine, opioid, serotonin, cortisol, oxytocin, etc.) which are related to the perception of pleasure, reward and motivation, but also to stress and arousal level, to the immune system and even to one's social attitude (Chanda and Levitin, 2013).

The purposes of this article are to define the basic characteristics that therapeutic interventions with music have in common and to categorize the types of intervention based on the use of music. This is of paramount importance to identify therapeutic interventions with music as distinguished from a general practice or fruition of it (Raglio, 2011). The latter, while producing beneficial effects on the individual, it does not possess the characteristics that define a therapeutic intervention. In many contexts in which the therapeutic use of music is put into practice, we can observe a considerable heterogeneity of interventions (Gold, 2009), a low level of definition of the therapeutic proposals and some methodological weaknesses in the evaluation of the effectiveness of such interventions.

There are some basic conditions that characterize a therapeutic intervention such as the presence of a qualified professional, a reference model that defines the theoretical and practical details which support the intervention (the therapy rationale), the presence of a therapeutic setting, and also the ability to define targets and therapeutic strategies of intervention with adequate awareness and the possibility to verify the therapeutic results achieved.

From a careful analysis of the scientific literature different applications that make use of music can be identified: music therapy interventions (following relational or rehabilitative models), music listening (individualized music listening or listening to music based on “music medicine” approach) and general music-based interventions.

Music therapy interventions in the scientific culture of the discipline are characterized by a relational component, which is considered essential, and by the presence of a qualified music therapist. In these cases, the treatment is therefore mediated by the presence of a therapist that uses applicative models based on psychological and/or neuroscientific theories. In the first case the reference is to the active techniques (which are based on a direct interactions with the patient/client using chiefly musical improvisation) and receptive ones (which involve listening to music in order to verbally elaborate the emotional content emerging from it) that aim at reducing psychological symptoms or complications arising therefrom, and at increasing relational and communication skills (Gold et al., 2009). Neuroscientific models are mainly based on the use of active techniques, such as music exercises (with a frequent use of rhythm), that constitute an effective motor, cognitive and sensory rehabilitation tool (the most common example in the literature is given by Neurologic Music Therapy) (Thaut, 2005).

Applications that involve listening to music can be divided into those where the patient listens to his favorite music (individualized music listening) (Särkämö et al., 2008; Gerdner, 2012), and those that go under the name of “music medicine” approach (in relation to the possibility of acting on specific symptoms or diseases) (Haas and Brandes, 2009). Listening to music is different from music therapy techniques mainly in that it does not imply a relational component between the patient/client and the therapist. A therapeutic value is therefore attributed to the mere action of music. In this case, the role of the music therapist is to lay down a program of music listening. This task can be carried out either according to the choices made by the patient/client, or according to the structural characteristics of music and its parameters in relation to the objective of the treatment. In the case of individualized music listening, a trained music therapist prepares a play-list containing pieces that are emotionally relevant for the patient/client, or pieces that meet his taste. Thus, the tracks are selected on the basis of indications given by the patient/client himself and through an anamnestic work involving formal or informal caregivers. Music listening based on the selection of favorite music, as documented in the literature, is aimed at people with a disease, often chronic or degenerative, in order to stimulate the patient/client cognitively and to reduce psychological or behavioral disturbances. In the case of music listening based on the “music medicine” approach, pieces are identified on the basis of the structural characteristics of music and their parameters, depending on the objective of the treatment. Tracks are then proposed that, because of their nature, can act on the patient/client by adjusting its physiological and psychological parameters passing from one phase of resonance to one of gradual change and adaptation that goes in the opposite direction to that of the pathology. Sometimes “music medicine” approach is aimed at reducing symptoms, sometimes momentarily, and therefore at having an immediate impact on the person. Frequently listening to music is used when the condition of the patient/client does not allow a direct interaction, or when logistical issues do not allow to set up a proper music therapy setting in the place where the treatment is performed (e.g., in hospitals).

The generic music-based interventions can be considered a non-specific use of music. In these, a professional with music skills organizes activities aimed at increasing the person's well-being. In particular, the objective is to improve the person's mood and motivation, promote socialization and stimulate sensory, motor and cognitive aspects in general. These are activities that lack a therapeutic rationale, they are usually performed in groups, where there is neither a real therapeutic setting nor intervention strategies aimed at achieving specific targets. The proposed activities are generally structured and consist of musical interaction (e.g., rhythmic accompaniment of a song, singing, movements associated with music, etc.), but can also be listening experiences in which the music turns out to be an excuse to stimulate verbalization, memories or to encourage of relaxation.

In Table 1 the identification of application areas in relation to the characteristics of the interventions is proposed.

**Table 1:** **Types of intervention with music in clinical settings and their characteristics**.

**Music therapy approaches**		**Listening to music approaches**		**Music-based** **approaches**
**Relational music therapy**	**Rehabilitative music therapy**	**Individualized music listening**	**Listening to music based on music medicine approach**	**General music-based approaches**
- Trained music therapist - Therapeutic Setting - Psychological models - Relationship as the core of intervention - Specific techniques: active approaches (in particular sonorous-musical improvisation) or receptive approaches - Aims (aspiring to become stable and long-lasting over time): attenuation of behavioral and psychiatric symptoms and prevention/stabilization of complications; increase in communication and relationship skills (sometimes improvement of cognitive and motor functions) - Presence of assessment criteria	- Trained music therapist -Rehabilitative setting -Neuroscientific models - Motor, cognitive and sensory rehabilitation as the core of intervention - Specific techniques: active approaches; exercises using sonorous-musical elements (in particular rhythm) - Aims (aspiring to become stable and long-lasting over time): motor, cognitive and sensory changes (sometimes psychological changes) - Presence of assessment criteria	- Trained music therapist helps patient/client to create a playlist including music that meets his/her taste - Absence of a specific therapeutic setting - Neuroscientific and psychological models - Self administration of listening to favorite music is the core of intervention - Aims: attenuation of behavioral and psychological symptoms; improvement of cognitive functions - Presence of assessment criteria	- A staff with medical/therapeutic background (sometimes supported by a music therapist) creates specific music listening programs for patient/client - Absence of a specific therapeutic setting - Physiological and psychological models - Self administration of listening to tailored music is the core of intervention - Aims: to balance and regulate physical and biological processes; to reduce physical and psychological symptoms (i.e., depression, anxiety, stress, sleeping disorders, hypertension, burnout, etc); psychological empowerment - Presence of assessment criteria	- Absence of a music therapist - Absence of a specific therapeutic setting - Absence of a specific intervention model - Making music (structured musical initiatives: rhythmic use of instruments, singing, movement associated to music, etc.) and listening to music (classical music, soothing music, evocative music, etc) - Aims: well-being, improving mood and motivation, promoting socialization, motor and cognitive stimulation, etc. - Presence of assessment criteria

From the literature analysis some key points and recommendations emerge that can promote the development of music therapy: first of all the need to use appropriate research methodologies to assess the effectiveness of interventions, focusing on randomized controlled trials and controlled clinical trials; moreover the need to define the interventions (Robb et al., 2011) and their application methods more adequately and finally the need to implement evidence-based approaches and research programs that would endorse the therapeutic results arising from the use of music in different clinical settings.

This may facilitate the integration of music interventions in hospitals and institutions (even in the many countries in which music therapy is not yet formally recognized) by promoting the use and spread of these effective non-pharmacological approaches, which are indeed applicable in several areas of health care, at low cost and without side effects.

## Conflict of interest statement

The authors declare that the research was conducted in the absence of any commercial or financial relationships that could be construed as a potential conflict of interest.
